# 1,2-Bis(2,4,6-trinitro­phen­yl)ethane

**DOI:** 10.1107/S1600536811042802

**Published:** 2011-10-29

**Authors:** Wen-Yan Wang, Ying Diao, Zhi-Hua Wei, Jian-Long Wang

**Affiliations:** a School of Chemical Engineering and Environment, North University of China, Taiyuan, People’s Republic of China

## Abstract

The title compound, C_14_H_8_N_6_O_12_, is centrosymmetric, the mid-point of the central C—C bond being located on an inversion centre. Two of the three independent nitro groups are disordered over two sites, with a site-occupancy ratio of 0.513 (3):0.487 (3). Weak inter­molecular C—H⋯O hydrogen bonding is present in the crystal structure.

## Related literature

For the synthesis of the title compound, see: Shipp (1964 ▶); Gilbert & Morristown (1980 ▶).
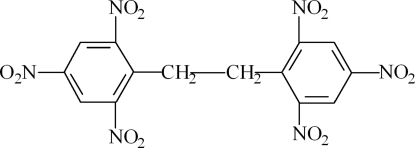


## Experimental

### 

#### Crystal data

C_14_H_8_N_6_O_12_
*M* *_r_* = 452.26
Monoclinic, 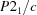

*a* = 5.8468 (5) Å
*b* = 8.1253 (11) Å
*c* = 17.977 (2) Å
β = 97.154 (8)°
*V* = 847.38 (17) Å^3^
*Z* = 2
Mo *K*α radiation
μ = 0.16 mm^−1^
*T* = 113 K
0.22 × 0.20 × 0.16 mm

#### Data collection

Rigaku Saturn724 CCD diffractometer
Absorption correction: multi-scan (*CrystalClear*; Rigaku/MSC, 2000 ▶) *T*_min_ = 0.966, *T*_max_ = 0.975
7531 measured reflections
2013 independent reflections
1503 reflections with *I* > 2σ(*I*)
*R*_int_ = 0.034

#### Refinement

*R*[*F* ^2^ > 2σ(*F* ^2^)] = 0.034
*wR*(*F* ^2^) = 0.099
*S* = 1.04
2013 reflections
186 parameters
70 restraints
H-atom parameters constrained
Δρ_max_ = 0.26 e Å^−3^
Δρ_min_ = −0.25 e Å^−3^

### 

Data collection: *CrystalClear* (Rigaku/MSC, 2000 ▶); cell refinement: *CrystalClear*; data reduction: *CrystalClear*; program(s) used to solve structure: *SHELXTL* (Sheldrick, 2008 ▶); program(s) used to refine structure: *SHELXTL*; molecular graphics: *SHELXTL*; software used to prepare material for publication: *SHELXTL*.

## Supplementary Material

Crystal structure: contains datablock(s) I, global. DOI: 10.1107/S1600536811042802/xu5348sup1.cif

Structure factors: contains datablock(s) I. DOI: 10.1107/S1600536811042802/xu5348Isup2.hkl

Supplementary material file. DOI: 10.1107/S1600536811042802/xu5348Isup3.cml

Additional supplementary materials:  crystallographic information; 3D view; checkCIF report

## Figures and Tables

**Table 1:** Hydrogen-bond geometry (Å, °)

*D*—H⋯*A*	*D*—H	H⋯*A*	*D*⋯*A*	*D*—H⋯*A*
C1—H1*A*⋯O4^i^	0.99	2.43	3.3669 (15)	158
C1—H1*B*⋯O5^ii^	0.99	2.37	3.147 (2)	134

Symmetry codes: (i) 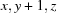; (ii) 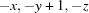.

**Fig. 1.**
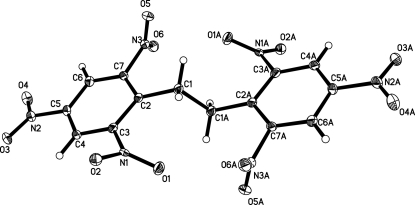
The molecular structure of the title compound with the atom numbering scheme. Displacement ellipsoids are drawn at the 30% probability level. H atoms are presented as a small spheres of arbitrary radius.

**Fig. 2.**
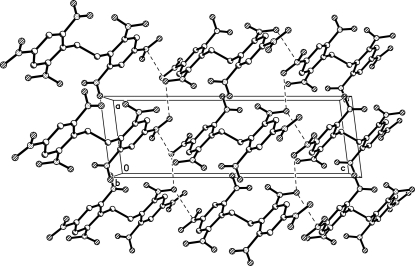
The crystal packing of the title compound.

## supplementary crystallographic information

### Comment

2,2',4,4',6,6'-Hexanitrostilbene is one of the most important heat resistant explosives. It can be prepared by treating the solution of TNT in tetrahydrofuran–methanol mixture with 5% sodium hypochlorite (Shipp, 1964). Later on its synthesis method was improved by Gilbert & Morristown (1980). As an intermediate, 2,2',4,4',6,6'-hexanitrobibenzyl was synthesized by the oxidation of TNT. Here we report the crystal structure of the title compound.

In the crystal structure, there is an inversion center in the molecule. Weak intermolecular C—H···O hydrogen bonding is present in the crystal structure.

### Experimental

The title compound was prepared according to literature method (Gilbert & Morristown, 1980). Single crystals were obtained by evaporation of a solution of the title compound in acetone at room temperature.

### Refinement

N1-Nitro and N3-nitro groups are disordered over two sites, occupancy ratio was refined to 0.513 (3):0.487 (3). For the disordered components, thermal parameters of the primed atoms were set to those of the unprimed ones, and all anisotropic thermal parameters were restrained to be nearly isotropic. The N—O distances were restrained to within 0.01 Å in the N1-nitro and N3-nitro groups. H atoms were positioned geometrically with C—H = 0.95 Å for benzene ring H and 0.99 Å for methylene H atoms, refined in riding mode with *U*_iso_(H) = 1.2*U*_eq_(C).

### Crystal data

**Table:**

C_14_H_8_N_6_O_12_	*F*(000) = 460
*M**_r_* = 452.26	*D*_x_ = 1.773 Mg m^−^^3^
Monoclinic, *P*2_1_/*c*	Mo *K*α radiation, λ = 0.71073 Å
Hall symbol: -P 2ybc	Cell parameters from 3228 reflections
*a* = 5.8468 (5) Å	θ = 2.3–27.9°
*b* = 8.1253 (11) Å	µ = 0.16 mm^−^^1^
*c* = 17.977 (2) Å	*T* = 113 K
β = 97.154 (8)°	Prism, colourless
*V* = 847.38 (17) Å^3^	0.22 × 0.20 × 0.16 mm
*Z* = 2	

### Data collection

**Table:**

Rigaku Saturn724 CCD diffractometer	2013 independent reflections
Radiation source: rotating anode	1503 reflections with *I* > 2σ(*I*)
multilayer	*R*_int_ = 0.034
Detector resolution: 14.22 pixels mm^-1^	θ_max_ = 27.9°, θ_min_ = 2.3°
ω and φ scans	*h* = −7→7
Absorption correction: multi-scan (*CrystalClear*; Rigaku/MSC, 2000)	*k* = −8→10
*T*_min_ = 0.966, *T*_max_ = 0.975	*l* = −23→23
7531 measured reflections	

### Refinement

**Table:**

Refinement on *F*^2^	Secondary atom site location: difference Fourier map
Least-squares matrix: full	Hydrogen site location: inferred from neighbouring sites
*R*[*F*^2^ > 2σ(*F*^2^)] = 0.034	H-atom parameters constrained
*wR*(*F*^2^) = 0.099	*w* = 1/[σ^2^(*F*_o_^2^) + (0.0569*P*)^2^ + 0.0176*P*] where *P* = (*F*_o_^2^ + 2*F*_c_^2^)/3
*S* = 1.04	(Δ/σ)_max_ < 0.001
2013 reflections	Δρ_max_ = 0.26 e Å^−^^3^
186 parameters	Δρ_min_ = −0.25 e Å^−^^3^
70 restraints	Extinction correction: *SHELXTL* (Sheldrick, 2008), Fc^*^=kFc[1+0.001xFc^2^λ^3^/sin(2θ)]^-1/4^
Primary atom site location: structure-invariant direct methods	Extinction coefficient: 0.025 (5)

### Special details

**Table:**

Geometry. All e.s.d.'s (except the e.s.d. in the dihedral angle between two l.s. planes) are estimated using the full covariance matrix. The cell e.s.d.'s are taken into account individually in the estimation of e.s.d.'s in distances, angles and torsion angles; correlations between e.s.d.'s in cell parameters are only used when they are defined by crystal symmetry. An approximate (isotropic) treatment of cell e.s.d.'s is used for estimating e.s.d.'s involving l.s. planes.
Refinement. Refinement of *F*^2^ against ALL reflections. The weighted *R*-factor *wR* and goodness of fit *S* are based on *F*^2^, conventional *R*-factors *R* are based on *F*, with *F* set to zero for negative *F*^2^. The threshold expression of *F*^2^ > σ(*F*^2^) is used only for calculating *R*-factors(gt) *etc*. and is not relevant to the choice of reflections for refinement. *R*-factors based on *F*^2^ are statistically about twice as large as those based on *F*, and *R*-factors based on ALL data will be even larger.

### Fractional atomic coordinates and isotropic or equivalent isotropic displacement parameters (Å^2^)

**Table:**

	*x*	*y*	*z*	*U*_iso_*/*U*_eq_	Occ. (<1)
N1	0.788 (2)	0.4687 (13)	0.1645 (5)	0.0296 (16)	0.513 (3)
O1	0.8771 (5)	0.5628 (3)	0.12216 (13)	0.0302 (6)	0.513 (3)
O2	0.815 (3)	0.489 (2)	0.2328 (6)	0.0260 (15)	0.513 (3)
N1'	0.824 (2)	0.4472 (14)	0.1631 (5)	0.0296 (16)	0.487 (3)
O1'	0.9430 (5)	0.4872 (4)	0.11433 (14)	0.0355 (7)	0.487 (3)
O2'	0.822 (3)	0.503 (2)	0.2263 (7)	0.035 (3)	0.487 (3)
O3	0.72410 (17)	−0.11091 (14)	0.26187 (5)	0.0387 (3)	
O4	0.48405 (19)	−0.23368 (11)	0.17726 (5)	0.0344 (3)	
N3	0.15225 (18)	0.19328 (12)	0.00293 (5)	0.0229 (3)	
O5	−0.0105 (3)	0.2748 (3)	0.01446 (10)	0.0295 (6)	0.513 (3)
O6	0.1555 (3)	0.0949 (3)	−0.04988 (9)	0.0301 (7)	0.513 (3)
O5'	−0.0438 (3)	0.1733 (4)	0.01768 (11)	0.0328 (6)	0.487 (3)
O6'	0.2022 (4)	0.2111 (3)	−0.06168 (10)	0.0345 (7)	0.487 (3)
N2	0.59258 (19)	−0.11271 (15)	0.20272 (6)	0.0299 (3)	
C1	0.4345 (2)	0.49440 (15)	0.03484 (6)	0.0222 (3)	
H1A	0.4797	0.5885	0.0685	0.027*	
H1B	0.2668	0.5029	0.0185	0.027*	
C2	0.4834 (2)	0.33521 (15)	0.07792 (6)	0.0226 (3)	
C3	0.6575 (2)	0.31668 (17)	0.13844 (6)	0.0267 (3)	
C4	0.6978 (2)	0.17462 (17)	0.18056 (6)	0.0271 (3)	
H4	0.8168	0.1692	0.2216	0.033*	
C5	0.5584 (2)	0.04194 (16)	0.16040 (7)	0.0263 (3)	
C6	0.3810 (2)	0.04812 (17)	0.10198 (7)	0.0268 (3)	
H6	0.2850	−0.0445	0.0892	0.032*	
C7	0.3490 (2)	0.19428 (16)	0.06306 (6)	0.0229 (3)	

### Atomic displacement parameters (Å^2^)

**Table:**

	*U*^11^	*U*^22^	*U*^33^	*U*^12^	*U*^13^	*U*^23^
N1	0.011 (3)	0.047 (2)	0.0282 (8)	−0.005 (2)	−0.0062 (11)	0.0187 (11)
O1	0.0339 (14)	0.0303 (14)	0.0260 (10)	−0.0121 (10)	0.0020 (9)	0.0048 (10)
O2	0.027 (3)	0.024 (3)	0.026 (2)	−0.0047 (19)	0.001 (2)	−0.002 (3)
N1'	0.011 (3)	0.047 (2)	0.0282 (8)	−0.005 (2)	−0.0062 (11)	0.0187 (11)
O1'	0.0315 (16)	0.0473 (18)	0.0260 (12)	−0.0210 (12)	−0.0031 (10)	0.0114 (12)
O2'	0.028 (3)	0.031 (4)	0.048 (5)	−0.004 (2)	0.010 (3)	0.008 (2)
O3	0.0289 (6)	0.0549 (7)	0.0298 (5)	−0.0031 (5)	−0.0069 (4)	0.0208 (5)
O4	0.0452 (7)	0.0309 (5)	0.0262 (5)	0.0059 (4)	0.0002 (4)	0.0024 (4)
N3	0.0192 (6)	0.0277 (6)	0.0213 (5)	−0.0009 (4)	0.0011 (4)	0.0056 (4)
O5	0.0182 (10)	0.0352 (14)	0.0345 (10)	0.0056 (9)	0.0009 (8)	0.0059 (9)
O6	0.0282 (11)	0.0410 (15)	0.0203 (9)	−0.0031 (9)	0.0001 (7)	−0.0006 (8)
O5'	0.0170 (11)	0.0421 (16)	0.0390 (12)	0.0004 (10)	0.0016 (9)	0.0030 (11)
O6'	0.0379 (13)	0.0474 (16)	0.0169 (9)	−0.0111 (10)	−0.0022 (8)	0.0054 (9)
N2	0.0254 (6)	0.0418 (7)	0.0225 (5)	0.0051 (5)	0.0030 (4)	0.0104 (5)
C1	0.0214 (7)	0.0296 (6)	0.0155 (5)	−0.0056 (5)	0.0013 (5)	0.0023 (5)
C2	0.0172 (6)	0.0361 (7)	0.0151 (5)	−0.0018 (5)	0.0039 (4)	0.0053 (5)
C3	0.0194 (7)	0.0426 (8)	0.0182 (6)	−0.0075 (5)	0.0026 (5)	0.0063 (5)
C4	0.0170 (7)	0.0473 (8)	0.0166 (6)	−0.0013 (5)	0.0003 (5)	0.0089 (5)
C5	0.0235 (7)	0.0370 (7)	0.0186 (6)	0.0023 (6)	0.0033 (5)	0.0108 (5)
C6	0.0235 (7)	0.0347 (7)	0.0220 (6)	−0.0040 (5)	0.0016 (5)	0.0066 (5)
C7	0.0162 (6)	0.0371 (7)	0.0149 (5)	−0.0006 (5)	0.0004 (4)	0.0052 (5)

### Geometric parameters (Å, °)

**Table:**

N1—O2	1.231 (8)	N2—C5	1.4697 (16)
N1—O1	1.238 (7)	C1—C2	1.5164 (16)
N1—C3	1.496 (7)	C1—C1^i^	1.550 (2)
N1'—O2'	1.226 (8)	C1—H1A	0.9900
N1'—O1'	1.228 (8)	C1—H1B	0.9900
N1'—C3	1.471 (7)	C2—C7	1.3957 (17)
O3—N2	1.2323 (13)	C2—C3	1.4024 (16)
O4—N2	1.2271 (15)	C3—C4	1.3846 (18)
N3—O5	1.199 (2)	C4—C5	1.3728 (19)
N3—O5'	1.219 (2)	C4—H4	0.9500
N3—O6'	1.241 (2)	C5—C6	1.3820 (17)
N3—O6	1.243 (2)	C6—C7	1.3792 (18)
N3—C7	1.4772 (14)	C6—H6	0.9500
			
O2—N1—O1	121.2 (11)	C1^i^—C1—H1B	109.1
O2—N1—C3	114.9 (9)	H1A—C1—H1B	107.8
O1—N1—C3	123.7 (6)	C7—C2—C3	113.41 (11)
O2'—N1'—O1'	129.5 (12)	C7—C2—C1	122.43 (10)
O2'—N1'—C3	117.6 (10)	C3—C2—C1	124.07 (11)
O1'—N1'—C3	112.9 (6)	C4—C3—C2	124.86 (12)
O5—N3—O5'	41.21 (12)	C4—C3—N1'	112.0 (6)
O5—N3—O6'	112.46 (16)	C2—C3—N1'	123.1 (6)
O5'—N3—O6'	123.81 (15)	C4—C3—N1	118.2 (5)
O5—N3—O6	125.15 (16)	C2—C3—N1	116.5 (5)
O5'—N3—O6	100.65 (16)	N1'—C3—N1	10.7 (11)
O6'—N3—O6	48.11 (12)	C5—C4—C3	117.07 (11)
O5—N3—C7	115.66 (13)	C5—C4—H4	121.5
O5'—N3—C7	120.60 (13)	C3—C4—H4	121.5
O6'—N3—C7	115.58 (13)	C4—C5—C6	122.48 (11)
O6—N3—C7	118.54 (12)	C4—C5—N2	119.78 (11)
O4—N2—O3	124.71 (11)	C6—C5—N2	117.73 (12)
O4—N2—C5	117.46 (10)	C7—C6—C5	117.28 (12)
O3—N2—C5	117.81 (11)	C7—C6—H6	121.4
C2—C1—C1^i^	112.47 (13)	C5—C6—H6	121.4
C2—C1—H1A	109.1	C6—C7—C2	124.88 (11)
C1^i^—C1—H1A	109.1	C6—C7—N3	114.24 (11)
C2—C1—H1B	109.1	C2—C7—N3	120.87 (10)
			
C1^i^—C1—C2—C7	−90.86 (16)	C3—C4—C5—C6	−1.5 (2)
C1^i^—C1—C2—C3	92.64 (16)	C3—C4—C5—N2	179.85 (11)
C7—C2—C3—C4	0.13 (18)	O4—N2—C5—C4	−171.13 (12)
C1—C2—C3—C4	176.91 (12)	O3—N2—C5—C4	10.35 (18)
C7—C2—C3—N1'	178.3 (5)	O4—N2—C5—C6	10.15 (18)
C1—C2—C3—N1'	−4.9 (5)	O3—N2—C5—C6	−168.36 (12)
C7—C2—C3—N1	−172.0 (4)	C4—C5—C6—C7	0.7 (2)
C1—C2—C3—N1	4.8 (5)	N2—C5—C6—C7	179.42 (11)
O2'—N1'—C3—C4	−64.8 (15)	C5—C6—C7—C2	0.6 (2)
O1'—N1'—C3—C4	117.4 (9)	C5—C6—C7—N3	−178.30 (11)
O2'—N1'—C3—C2	116.8 (13)	C3—C2—C7—C6	−0.98 (18)
O1'—N1'—C3—C2	−61.0 (13)	C1—C2—C7—C6	−177.82 (12)
O2'—N1'—C3—N1	62 (4)	C3—C2—C7—N3	177.83 (10)
O1'—N1'—C3—N1	−116 (5)	C1—C2—C7—N3	0.99 (18)
O2—N1—C3—C4	−38.7 (14)	O5—N3—C7—C6	109.24 (19)
O1—N1—C3—C4	136.6 (10)	O5'—N3—C7—C6	62.5 (2)
O2—N1—C3—C2	134.0 (11)	O6'—N3—C7—C6	−116.35 (17)
O1—N1—C3—C2	−50.7 (13)	O6—N3—C7—C6	−61.96 (18)
O2—N1—C3—N1'	−96 (4)	O5—N3—C7—C2	−69.7 (2)
O1—N1—C3—N1'	79 (3)	O5'—N3—C7—C2	−116.4 (2)
C2—C3—C4—C5	1.1 (2)	O6'—N3—C7—C2	64.72 (19)
N1'—C3—C4—C5	−177.3 (5)	O6—N3—C7—C2	119.11 (17)
N1—C3—C4—C5	173.0 (5)		

Symmetry codes: (i) −*x*+1, −*y*+1, −*z*.

### Hydrogen-bond geometry (Å, °)

**Table:**

*D*—H···*A*	*D*—H	H···*A*	*D*···*A*	*D*—H···*A*
C1—H1A···O4^ii^	0.99	2.43	3.3669 (15)	158
C1—H1B···O5^iii^	0.99	2.37	3.147 (2)	134

Symmetry codes: (ii) *x*, *y*+1, *z*; (iii) −*x*, −*y*+1, −*z*.
